# Genetic and phenotypic parameters for subclinical mastitis occurrence in pasture-based Girolando cows from a research herd in Southeast Brazil

**DOI:** 10.3168/jdsc.2025-0966

**Published:** 2026-02-19

**Authors:** Gabriel C. Medeiros, Camila S. Mussi, Abdulhakeem B. Ajibike, Bruno Balduino Berber Freitas, Fernando Baldi, Elisângela C.M. Oliveira, José Bento S. Ferraz, Cleber Barbosa de Oliveira

**Affiliations:** 1 Department of Veterinary Medicine, School of Animal Sciences and Food Engineering, University of São Paulo, Pirassununga, SP, 13635-900, Brazil; 2 Department of Animal Production Technology, Faculty of Animal and Fisheries Technology, Oyo State College of Agriculture and Technology, Igboora, OY, 201103, Nigeria; 3 Instituto Federal de Educação, Ciência e Tecnologia do Triângulo Mineiro–Campus Uberaba, Uberaba, MG, 38064-790, Brazil

## Abstract

Subclinical mastitis is still one of the most important health issues in dairy herds, leading to substantial economic losses due to reduced milk yield, treatment costs, and premature culling. Understanding the genetic basis of this disorder is essential in supporting selection strategies to enhance udder health and herd productivity. The aim of this study was to estimate the genetic and phenotypic parameters for subclinical mastitis occurrence using information from 748 lactations of 279 pasture-based Girolando cows from the Federal Institute of Triângulo Mineiro (IFTM)–Uberaba research herd, calved between 2012 and 2024. Variance components were obtained using a Bayesian approach under a complete animal model. The heritability estimates (posterior medians [highest posterior density interval]) for subclinical mastitis were 0.14 [0.09–0.20] and 0.15 [0.10–0.20] for the right (RF) and left (LF) front quarters, and 0.13 [0.08–0.18] and 0.15 [0.09–0.20] for the right (RR) and left (LR) rear quarters, respectively. The repeatabilities were 0.41 [0.37–0.45] and 0.35 [0.31–0.39] for RF and LF, and 0.35 [0.32–0.39] and 0.41 [0.37–0.45] for the RR and LR quarters, respectively. The low to moderate heritabilities indicate that subclinical mastitis is highly influenced by environmental factors, and the moderate repeatabilities estimates indicate that these traits will benefit from a more frequent data collection. The results suggest that the genetic progress for subclinical mastitis in pasture-based Girolando cows will be slow. Genetic and phenotypic correlations among quarters were also estimated using a multiple-trait model. Genetic correlations were consistently high, exceeding 0.80 for all quarter combinations, indicating a largely shared genetic background controlling susceptibility to subclinical mastitis across udder quarters. In contrast, phenotypic correlations were moderate ranging from 0.50 [0.47–0.54] between RF and LR, and 0.59 [0.56–0.62] between RF and LF, suggesting that environmental and management-related factors contribute more substantially to differences in mastitis occurrence among quarters than genetic effects. Overall, these results suggest that selection for reduced subclinical mastitis in one quarter is expected to result in favorable correlated responses in the others. However, given the limited dataset size, further studies using larger multiherd populations in tropical production systems, and genomic information, are necessary to validate these correlations and to investigate potential breed composition differences in udder and teat morphology that can affect mastitis susceptibility.

## Highlights

• Quarter-level data enable genetic evaluation of subclinical mastitis.
• Subclinical mastitis presented low heritability and moderate repeatability across udder quarters.
• Genetic progress for mastitis resistance in pasture-based cows is expected to be slow.

## Graphical Abstract

Subclinical mastitis is one of the main health and economic challenges in dairy production. This study investigated the genetic background of subclinical mastitis at the udder quarter level in Girolando cows using data from 748 lactations of 279 animals raised in a pasture-based system. Subclinical mastitis was assessed for each udder quarter using the California Mastitis Test, and genetic parameters were estimated through a Bayesian animal model. Heritability estimates ranged from 0.13 to 0.15, and repeatabilities from 0.35 to 0.41 across the right and left, front and rear quarters. The quarter-level analysis revealed consistent patterns of genetic and environmental variation among quarters. Low heritabilities indicate strong environmental influence, whereas the moderate repeatabilities suggest benefits from frequent and consistent data recording. These findings indicate that subclinical mastitis can be genetically evaluated based on quarter-level data, although genetic progress for resistance is expected to be slow. h2 = heritability; RF = right front quarter; LF = left front quarter; RR = right rear quarter; LR = left rear quarter; t = repeatability; un = unit.

**Figure:**
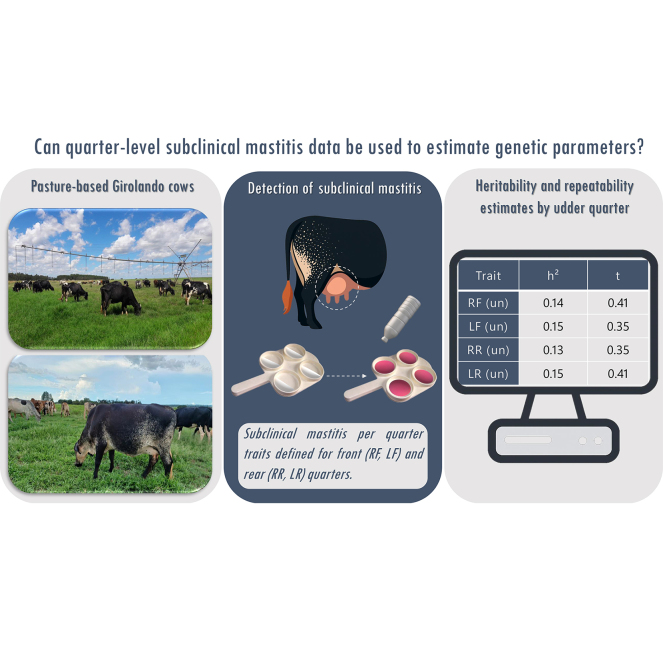

Mastitis is defined as an inflammatory process of the mammary gland, most often of bacterial origin, manifesting in either clinical or subclinical forms (Natzke, 1981). Clinical mastitis is characterized by visible signs of inflammation, whereas subclinical mastitis requires specific diagnostic methods, such as SCC and the California Mastitis Test (**CMT**; Coldebella et al., 2003; Zhao and Lacasse, 2008). Mastitis is a disease of high relevance in dairy cattle, as it compromises herd productivity and milk quality, resulting in economic losses. Early diagnosis is therefore critical for effective management and control. According to Rodrigues et al. (2018), subclinical mastitis requires special attention because it does not present obvious clinical signs, making detection more difficult and amplifying its negative impact on production.

The mammary gland is the primary source of mastitis-causing agents. Transmission of microorganisms to other animals in the herd occurs during milking and through environmental exposure. Proper hygiene and disinfection of the environment, animals, personnel, and milking equipment are essential to reduce its occurrence (Philpot and Nickerson, 2002) as the incidence of mastitis is associated with insufficient or poor hygienic management before, during, and after milking. Selecting genetically resistant animals to mastitis is an effective strategy to reduce disease incidence, improve animal welfare, and decrease production costs in dairy systems.

Genetic evaluations of mastitis resistance are conducted based on SCC, as this trait shows a high genetic correlation with disease occurrence (Urioste et al., 2011). Although SCC typically exhibits higher heritability compared with clinical mastitis, it is not routinely measured by tropical dairy producers because it requires specialized laboratory analyses. In contrast, clinical mastitis can be evaluated by producers during routine milking management (Oliveira et al., 2015). Nevertheless, selection based solely on SCC appears less effective than direct selection based on clinical mastitis (Heringstad et al., 2000). The mastitis predictive value of SCC depends on herd infection prevalence: In herds with high prevalence, SCC may be a good and reliable predictor, whereas in herds with low prevalence of mammary disease, it is less informative (Kehrli and Shuster, 1994). Both measurements can be incorporated into genetic evaluation models to enable a more precise assessments of genetic improvement for mastitis resistance (Oliveira et al., 2015). However, previous studies (Kehrli and Shuster, 1994; Heringstad et al., 2000; Urioste et al., 2011; Oliveira et al., 2015; Narayana et al., 2021) provide no evidence on the use of the CMT as a tool to measure subclinical mastitis in herds or its application in genetic improvement programs, highlighting a gap in literature. Addressing this gap is important, as CMT could offer a practical alternative for producers in resource-limited settings and improve the accuracy of genetic selection for mastitis resistance. Therefore, the objective of this study was to estimate the genetic and phenotypic parameters for subclinical mastitis occurrence in Girolando and Gir dairy cows.

Records from 748 lactations of 279 dairy cows (257 Girolando animals with varying genetic compositions [1/4, 3/8, 1/2, 5/8, and 3/4] and 22 purebred Gir cows calving between 2012 and 2025) were evaluated. The animals belonged to the Federal Institute of Triângulo Mineiro (**IFTM**)–Uberaba campus (Uberaba, Minas Gerais, Brazil). The herd was managed under an extensive *Urochloa* spp. pasture system, with free access to mineral salt. During the dry season, cows received supplementation based on corn silage and concentrate according to their production level. The cows were milked twice daily using a mechanized milking parlor with a herringbone system, and milk yield data were measured every 2 wk. Initially, the “strip cup test” was performed by discarding the first 3 to 5 streams of milk from each quarter into a black-bottomed cup to observe the presence of clots. After this, the CMT was conducted immediately, after hygienic preparation of the udder and removal of the first streams (Philpot and Nickerson, 1991), by immersing the teats in a pre-dipping solution, leaving them for 30 s, and then drying using disposable paper towels (one per teat). For the CMT, milk from each mammary quarter was collected in a cup of the CMT tray up to the first mark, followed by adding an equal volume of reagent prepared with commercial detergent as described by Langenegger et al. (1970) to the second mark, and stirred for ~30 s in circular movements. The reactions were interpreted with grading scores for 1 (no reaction), 2 (weakly positive), 3 (positive), to 4 (strongly positive) as described by Philpot and Nickerson (1991).

The CMT records for each quarter (right front [**RF**], left front [**LF**], right rear [**RR**], and left rear [**LR**]) were considered as 4 different traits. For each trait, phenotypes from animals with known pedigree, breed composition, and at least 10 records and at least 10 animals in each year-season of calving group were used for further analyses. The data editing and quality control of the phenotypic database were performed using R software 4.3.3 (R Core Team, 2025). The linear covariates of direct and maternal percentage of Holstein heterosis and recombination coefficients were fitted based on the available pedigree records, using the methodology proposed by Oliveira et al. (2024).

The variance components and genetic parameters for the traits were estimated using the GIBBSF90+ software (Misztal et al., 2024). The fitted model can be described as follows:

[1] **y** = **Xb** + **Zu** + **Wp** + **e**,

In this model, **y** represents the vector containing repeated phenotypic records for the analyzed trait, **b** is a vector containing the fixed effects of, age of the animal at data collection (linear and quadratic), direct percentage of Holstein breed, maternal percentage of Holstein breed, heterosis, recombination, lactation number (1, 2, 3, or 4+), and concatenation of year and season (April–September, October–March) of calving, and 5 classes of DIM (5–20, 21–50, 51–130, 131–230, 231–305). The vector **u** contains the additive genetic effects of the animals included in **y**, $u\;\left(0,G\sigma_{u}^{2}\right),$ where **G** represent the pedigree relationship matrix (**A**; Wright, 1922), $\sigma_{u}^{2}$ is the genetic additive variance; **p** is a vector containing the permanent environmental effects of the cows in each lactation in **y**, $pI\;\left(0,\sigma_{pe}^{2}\right),$ where **I** is an identity matrix and $\sigma_{pe}^{2}$ is the permanent environmental variance. The vector **e** contains the random residual effects, assumed to follow $e\;\left(0,I\sigma_{e}^{2}\right),$ where **I** is an identity matrix and $\sigma_{e}^{2}$ is the residual variance. **X**, **Z**, and **W** are matrices that link the phenotypic records to the fixed, additive genetic effects, and permanent environment effects, respectively.

A bivariate linear animal model was used to estimate the genetic and phenotypic correlations among all traits. The multiple-trait model included the same fixed and random effects described previously. Therefore, the genetic parameters were estimated using the following multiple-trait repeatability model:

$$\left(\begin{array}{c} y_{1}\\ y_{2} \end{array}\right)=\left(\begin{array}{cc} x_{2} & 0\\ 0 & x_{2} \end{array}\right)\left(\begin{array}{c} b_{1}\\ b_{2} \end{array}\right)+\left(\begin{array}{cc} z_{1} & 0\\ 0 & z_{2} \end{array}\right)\left(\begin{array}{c} u_{1}\\ u_{2} \end{array}\right)+\left(\begin{array}{cc} w_{1} & 0\\ 0 & w_{2} \end{array}\right)\left(\begin{array}{c} p_{1}\\ p_{2} \end{array}\right)+\left(\begin{array}{c} e_{1}\\ e_{2} \end{array}\right),$$

where **y**_1_ and **y**_2_ contain repeated records of traits 1 and 2; **x**_1_ and **x**_2_ are incidence matrices relating observations to the vectors of fixed effects **b**_1_ and **b**_2_ for traits 1 and 2, respectively; **z**_1_ and **z**_2_ are incidence matrices relating observations to the vectors of additive genetic effects **u**_1_ and **u**_2_; **w**_1_ and **w**_2_ are incidence matrices relating observations to the vectors of permanent environmental effects **p**_1_ and **p**_2_; **u**_1_ and **u**_2_ are the vectors of additive genetic effects for traits 1 and 2, respectively; **b**_1_ and **b**_2_ are the vectors containing the fixed effects for both traits, which are the same as described for the single-trait analyses; **p**_1_ and **p**_2_ are the vectors containing the permanent environmental effects of the cows in **y**_1_ and **y**_2_; and **e**_1_ and **e**_2_ are the vectors containing the random residual effects. We assumed that E(**y**) = **Xb**; Var(**a**) = **G** × **Sa**; Var(**p**) = **I** × **Sp**; and Var(**e**) = **I** × **Se**, where **Sa** is the additive genetic covariance matrix; **Sp** is the permanent environmental covariance matrix; **Se** is the residual covariance matrix; **G** is the pedigree relationship matrix; **I** is an identity matrix; and × represents the direct product of the matrices. The vectors **a**, **p**, and **e** were assumed to be uncorrelated. The analyses were performed using the GIBBSF90+ software (Misztal et al., 2024). A total of 600,000 samples were generated, with the first 100,000 discarded as burn-in. From the remaining 500,000 samples, every 100th iteration was retained for posterior analysis. Convergence diagnostics were assessed using the Geweke test (Geweke, 1992).

Records related to subclinical mastitis for 748 lactations of 279 animals are presented in Table 1. Differences in the number of records between the 4 quarters were due to injuries or loss of quarters (or both) during data collection. The similar patterns across quarters indicate that milking management and conditions were uniform for all animals, with no evidence of localized contamination or trauma on a single side of the udder. The low average score of ~1.5 suggests that most of the herd had good udder health, with few cases of subclinical mastitis. However, the high CV (62%–63%) indicates heterogeneity in the mastitis scores, suggesting the presence of subgroups of cows with elevated scores (3–4) that may require individual monitoring. This study provides the first report of SD and CV for subclinical mastitis scores, as previous research has not reported such for quarter-specific scores in an individual quarter analysis (anterior vs. posterior; right vs. left) using the CMT.

**Table 1:** Descriptive statistics of California Mastitis Test traits in pasture-based Girolando cows from the IFTM–Uberaba research herd^1^

Trait	Number of animals	Number of records	Mean	SD	Minimum	Maximum	CV (%)
RF	279	5,775	1.56	0.99	1	4	63.1
LF	279	5,773	1.54	0.97	1	4	63.0
RR	279	5,779	1.52	0.95	1	4	62.2
LR	279	5,779	1.54	0.97	1	4	63.2

1 RF = right front quarter; LF = left front quarter; RR = right rear quarter; LR = left rear quarter.

The variance component estimates (posterior median and highest posterior density [**HPD**] interval) for the 4 quarters are presented in Table 2, and low heritabilities (0.13–0.15) and moderate repeatabilities (0.35–0.41) were recorded, suggesting a limited potential response to genetic selection for this trait. The low heritability shows that most observed phenotypic variation is attributable to environmental rather than genetic factors, hence, restricting the expected progress through genetic or genomic selection. Nevertheless, the heritabilities and repeatabilities values obtained in this study were higher than heritabilities of 0.11 and 0.047, and repeatabilities of 0.21 and 0.40 reported by Oliveira et al. (2015) and Narayana et al. (2021), respectively. However, the observed values were in close agreement with an estimated posterior median of 0.10 (HPD = 0.02–0.22) for SCC in pasture-based dairy cows in Chile by Uribe et al. (2022). Although the trait exhibited low additive genetic control, our findings suggest that favorable management and environmental conditions in this herd may have favored the high expression of additive genetic variability, resulting in higher genetic parameter estimates and higher phenotypic stability across lactations.

**Table 2:** Posterior medians and highest posterior density (HPD) intervals (in parentheses) of variance components, heritability, and repeatability for California Mastitis Test in pasture-based Girolando cows from the IFTM–Uberaba research herd^1^

Trait	σ^2^_*g*_	σ^2^_*pe*_	σ^2^_*e*_	σ^2^_*p*_	h^2^	t
RF	0.13	0.24	0.54	0.91	0.14	0.41
	(0.08–0.19)	(0.20–0.29)	(0.52–0.56)	(0.85–0.97)	(0.09–0.20)	(0.37–0.45)
LF	0.14	0.18	0.57	0.89	0.15	0.35
	(0.08–0.19)	(0.14–0.21)	(0.55–0.60)	(0.83–0.94)	(0.10–0.20)	(0.31–0.39)
RR	0.11	0.19	0.54	0.84	0.13	0.35
	(0.07–0.16)	(0.15–0.22)	(0.52–0.56)	(0.79–0.89)	(0.08–0.18)	(0.32–0.39)
LR	0.14	0.24	0.55	0.93	0.15	0.41
	(0.08–0.20)	(0.20–0.28)	(0.53–0.57)	(0.87–0.99)	(0.09–0.20)	(0.37–0.45)

1 RF = right front quarter; LF = left front quarter; RR = right rear quarter; LR = left rear quarter; σ^2^_*g*_ = additive genetic variance; σ^2^_*pe*_ = permanent environmental variance; σ^2^_*e*_ = residual variance; σ^2^_*p*_ = phenotypic variance; t = repeatability.

Overall, the low heritability suggests that improvements through management may have a greater impact than genetic selection. However, differences among quarters indicate relevant anatomical variation, which could justify quarter-specific strategies, particularly for the LR, which combined higher repeatability and greater genetic variation. The high residual variance in all cases reflected a strong influence of uncontrolled factors, and this observation was consistent with the values reported by Oliveira et al. (2015).

The estimated phenotypic variance $\left(\sigma_{p}^{2}\right)$ ranged from 0.84 un^2^ (RR; **un** = units) to 0.93 un^2^ (LR), an indication of overall moderate variability among the quarters of cows in the IFTM dairy herd. This variation reflects a combination of genetic and environmental factors, with environmental effects (permanent and residual) predominating over genetic effects. The highest $\sigma_{p}^{2}$ observed in the LR (0.93 un^2^) suggests a greater phenotypic heterogeneity in this region, which could be associated with differential sensitivity to management and physiological factors. The RR showed the lowest phenotypic variance of 0.84 un^2^, indicating high uniformity among the animals. The LR exhibited the highest genetic variance (0.14 un^2^), highest permanent environmental effect (0.24 un^2^), and highest phenotypic variance (0.93 un^2^), highlighting the consistency across measurements. The LF presented similar heritability (0.15) but lower repeatability (0.35), suggesting lower stability across lactations. Although there is sufficient variability for selection, the results revealed a predominant environmental influence limiting the potential for direct genetic gain.

The pasture-based environment directly shapes both exposure to mastitis pathogens and the expression of udder health phenotypes (McDougall et al., 2009). Extensive grazing increases exposure to environmental stressors such as soil, fecal contamination, and wet pasture conditions, as well as the frequency of transition between the pasture paddocks and milking parlor, which enhances the prevalence of environmental pathogens over contagious ones, resulting in higher environmental variance in mastitis indicators (Stanek et al., 2024). At the same time, lower production intensity and reduced metabolic stress associated with grazing systems can improve innate immune resilience, which in turn affects the proportion of phenotypic variance attributable to genetics. Therefore, the observed low heritabilities and moderate repeatabilities should be interpreted in the context of pasture-based exposure patterns and management heterogeneity, and caution is warranted when extrapolating these parameters to confinement or high-input systems.

The genetic correlations are shown in the upper diagonal and phenotypic correlations in the lower diagonal of Table 3. To our knowledge, there are no previous reports in the literature evaluating genetic and phenotypic correlations for subclinical mastitis at the individual quarter level, which limits direct comparisons. Overall, the results in Table 3 indicate that environmental effects play a stronger role in shaping correlations among quarters than genetic effects, as evidenced by the consistently moderate phenotypic correlations contrasted with uniformly high genetic correlations, all exceeding 0.80. These results suggest a largely shared genetic architecture underlying susceptibility to subclinical mastitis across quarters. Notably, genetic correlations involving the LR were comparatively lower, although still high, which may reflect specific characteristics of the studied population. In the studied herd, the LR showed a higher proportion of mastitis in animals with higher Holstein composition (>62.5%), a group in which a greater incidence of mastitis was observed in this quarter relative to others. Given the relatively small dataset, this imbalance in breed composition may have introduced a slight distortion, contributing to the reduced genetic correlations involving the LR. These findings highlight the need for further validation of variance components using larger datasets from multiple herds and tropical production systems. Moreover, the inclusion of genomic information would be particularly valuable to assess whether genes identified as significant for mastitis resistance in one quarter can be extrapolated to others, as well as to investigate potential breed-related differences in teat morphology, including sphincter structure and teat length, between purebred Holstein cattle and indicine–taurine crossbred animals.

**Table 3:** Posterior medians and highest posterior density (HPD) intervals (in parentheses) for genetic (above the diagonal) and phenotypic (below the diagonal) correlations among California Mastitis Test (CMT) traits in pasture-based Girolando cows from the IFTM–Uberaba research herd^1^

Trait	RF	LF	RR	LR
RF	—	0.99	0.99	0.83
		(0.96–0.99)	(0.95–0.99)	(0.69–0.94)
LF	0.59	—	0.98	0.85
	(0.56–0.62)		(0.94–0.99)	(0.75–0.94)
RR	0.54	0.53	—	0.93
	(0.51–0.57)	(0.50–0.56)		(0.86–0.99)
LR	0.50	0.55	0.57	—
	(0.47–0.54)	(0.52–0.58)	(0.54–0.60)	

1 RF = right front quarter; LF = left front quarter; RR = right rear quarter; LR = left rear quarter.

This study has limitations that constrain the interpretation and external validity of the genetic parameter estimates. Records originate from a single research herd managed under a specific pasture-based protocol reduces the generalizability to other herds and production systems; the dataset (748 lactations from 279 cows) provides reasonable power but may be underpowered to detect small genetic effects or genotype-by-environment interactions. Subclinical mastitis status was assessed with the CMT rather than by repeated quarter-level SCC, milk electroconductivity, or bacteriological culture, which introduces potential misclassification and may inflate residual variance; differences in the number of records among quarters (due to teat injuries or loss) can also bias estimates. Finally, the large residual component reported indicates an unmeasured sources of environmental heterogeneity (seasonal management, temperature of the day) that may reduce the heritability estimates precision. As next steps, the genetic correlation between quarters, comparison of Spearman correlation between CMT and SCC, and other milk quality and production traits will be performed to enhance the predictive models and identify the correct ponderation for these traits in a selection index for these crossbred animals in pasture-based conditions.

This study provides novel estimates of genetic and phenotypic parameters for subclinical mastitis in Girolando cows managed under pasture-based conditions in tropical Brazil. Despite the moderate repeatabilities and low to moderate heritabilities obtained, the results confirm that additive genetic variance exists for mastitis resistance, supporting the inclusion feasibility of this trait in selection programs. The predominance of environmental effects highlights the strong influence of pasture management, hygiene practices, and seasonal variability on disease expression. Consequently, genetic improvement should be complemented with continued optimization of milking routines and environmental hygiene to effectively reduce mastitis incidence. Overall, these findings contribute valuable baseline information for the genetic evaluation of udder health in tropical pasture-based dairy systems and reinforce the importance of integrating both genetic and management strategies to enhance animal welfare and productivity in tropical crossbred dairy herds.

## References

[bib1] Coldebella A., Machado P.F., Demétrio C.G.B., Ribeiro P.J., Corassin C.H., Meyer P.M., Cassoli L.D. (2003). Contagem de células somáticas e produção de leite em vacas holandesas de alta produção (Somatic cells count and milk yield in high production Holstein cows). Pesqui. Agropecu. Bras..

[bib2] Geweke J., Berger J.O., Bernardo J.M., Dawid A.P., Smith A.F.M. (1992). Bayesian Statistics.

[bib3] Heringstad B., Klemetsdal G., Ruane J. (2000). Selection for mastitis resistance in dairy cattle: A review with focus on the situation in the Nordic countries. Livest. Prod. Sci..

[bib4] Kehrli M.E., Shuster D.E. (1994). Factors affecting milk somatic cells and their role in health of the bovine mammary gland. J. Dairy Sci..

[bib5] Langenegger J., Coelho N.M., Langenegger C.H., Castro R.P. (1970). Estudo da incidência da mastite bovina na bacia leiteira do Rio de Janeiro. Pesqui. Agropecu. Bras..

[bib6] McDougall S., Parker K.I., Heuer C., Compton C.W.R. (2009). A review of prevention and control of heifer mastitis via non-antibiotic strategies. Vet. Microbiol..

[bib7] Misztal I., Tsuruta S., Lourenco D.A.L., Masuda Y., Aguilar I., Legarra A., Vitezica Z. (2014). https://nce.ads.uga.edu/wiki/lib/exe/fetch.php?media=blupf90_all.pdf.

[bib8] Narayana S.G., Schenkel F., Miglior F., Chud T., Abdalla E.A., Naqvi S.A., Malchiodi F., Barkema H.W. (2021). Genetic analysis of pathogen-specific intramammary infections in dairy cows. J. Dairy Sci..

[bib9] Natzke R.P. (1981). Elements of mastitis control. J. Dairy Sci..

[bib10] Oliveira E.C.M., Medeiros G.C., Baldi F.S., Giacomini G., Núñez-Domínguez R., Eler J.P., Espigolan R., Gama L.T., Ferraz J.B.S. (2024). Genetic structure of the Montana Beef Composite, a program to increase beef production in tropical and subtropical environments. Genet. Mol. Res..

[bib11] Oliveira E.J., Bignardi A.B., Santana M.L., Paz C.C.P., Zadra L.E.F. (2015). Associação genética entre ocorrência de mastite clínica e produção de leite em vacas holandesas. Cienc. Rural.

[bib12] Philpot W.N., Nickerson S.C. (1991).

[bib13] Philpot W.N., Nickerson S.C. (2002).

[bib14] R Core Team. 2025. R: A Language and Environment for Statistical Computing. R Foundation for Statistical Computing, Vienna, Austria.

[bib15] Rodrigues T.P., Coelho M.G.A.P., Santos E.B., Costa I.S., Cortez M.A.S. (2018). Mastite bovina: Influência na produção, composição e rendimento industrial do leite e derivados. Arquivos de Pesquisa Animal..

[bib16] Stanek P., Żółkiewski P., Januś E. (2024). A review on mastitis in dairy cows research: Current status and future perspectives. Agriculture.

[bib17] Uribe H., Lembeye F., González H. (2022). Estimation of genetic parameters for subclinical mastitis using a threshold model in first-parity dairy cows under pasture-based systems of Los Ríos Region in Chile. Austral J. Vet. Sci..

[bib18] Urioste J.I., Pérez C., Rorato F., Neser M. (2011). Genetic variability of alternative somatic cell count traits and their relationship with clinical and subclinical mastitis. Interbull Bull..

[bib19] Wright S. (1922). Coefficients of inbreeding and relationship. Am. Nat..

[bib20] Zhao X., Lacasse P. (2008). Mammary tissue damage during bovine mastitis: Causes and control. J. Anim. Sci..

